# Enhancing full-length antibody production by signal peptide engineering

**DOI:** 10.1186/s12934-016-0445-3

**Published:** 2016-03-02

**Authors:** Yizhou Zhou, Peter Liu, Yutian Gan, Wendy Sandoval, Anand Kumar Katakam, Mike Reichelt, Linda Rangell, Dorothea Reilly

**Affiliations:** Department of Early Stage Cell Culture, Genentech Inc., 1 DNA way, South San Francisco, CA 94080 USA; Department of Protein Chemistry, Genentech Inc., 1 DNA way, South San Francisco, CA 94080 USA; Department of Pathology, Genentech Inc., 1 DNA way, South San Francisco, CA 94080 USA

**Keywords:** Monoclonal antibody, Signal peptide, *Escherichia coli*, Secretion, Protein production

## Abstract

**Background:**

Protein secretion to the periplasm of *Escherichia coli* offers an attractive route for producing heterologous proteins including antibodies. In this approach, a signal peptide is fused to the N-terminus of the heterologous protein. The signal peptide mediates translocation of the heterologous protein from the cytoplasm to the periplasm and is cleaved during the translocation process. It was previously shown that optimization of the translation initiation region (TIR) which overlaps with the nucleotide sequence of the signal sequence improves the production of heterologous proteins. Despite the progress, there is still room to improve yields using secretion as a means to produce protein complexes such as full-length monoclonal antibodies (mAbs).

**Results:**

In this study we identified the inefficient secretion of heavy chain as the limitation for full-length mAb accumulation in the periplasm. To improve heavy chain secretion we investigated the effects of various signal peptides at controlled TIR strengths. The signal peptide of disulfide oxidoreductase (DsbA) mediated more efficient secretion of heavy chain than the other signal peptides tested. Mutagenesis studies demonstrated that at controlled translational levels, hydrophobicity of the hydrophobic core (H-region) of the signal peptide is a critical factor for heavy chain secretion and full-length mAb accumulation in the periplasm. Increasing the hydrophobicity of a signal peptide enhanced heavy chain secretion and periplasmic levels of assembled full-length mAbs, while decreasing the hydrophobicity had the opposite effect.

**Conclusions:**

This study demonstrates that under similar translational strengths, the hydrophobicity of the signal peptide plays an important role in heavy chain secretion. Increasing the hydrophobicity of the H-region and controlling TIR strengths can serve as an approach to improve heavy chain secretion and full-length mAb production in *E. coli*.

**Electronic supplementary material:**

The online version of this article (doi:10.1186/s12934-016-0445-3) contains supplementary material, which is available to authorized users.

## Background

Protein secretion to the periplasm of *Escherichia coli* offers an attractive route to produce heterologous proteins that contain disulfide bonds [1–4]. In this approach, the N-terminus of the heterologous protein is fused to a signal peptide that mediates translocation of the protein from the cytoplasm to the periplasm. The signal peptide is cleaved during the translocation process. Compared to cytoplasmic accumulation, secretory production of heterologous proteins has several advantages. First, the native N-terminal amino acid of the heterologous protein is maintained after the signal peptide is cleaved. Second, the oxidizing environment and enzymes in the periplasm facilitate correct disulfide bond formation [4]. Moreover, low concentrations of endogenous proteins in the periplasm make it easier to isolate the heterologous protein from host protein contaminants at laboratory scale [1, 3, 5–7].

Despite the advantages, it is still in general challenging to use secretion as a means to produce some heterologous proteins, especially protein complexes such as full-length mAbs [3, 8]. Limitations include inefficient translocation of heterologous proteins from the cytoplasm to the periplasm and incomplete processing of the signal peptide [3, 7, 9, 10]. Unprocessed precursors tend to aggregate and form inclusion bodies in the cytoplasm [9, 11]. As a result, the yields of heterologous proteins in the periplasm are often reported to be low [1, 3].

To improve protein accumulation in the periplasm, extensive studies have focused on the primary structures of signal peptides. Signal peptides are commonly composed of three distinct regions: a charged N-terminal region, a hydrophobic core region often referred to as the H-region, and a C-terminal region recognized by the signal peptidase [12, 13]. A large body of literature using *E. coli* proteins or fusion proteins as cargo proteins suggests that increasing the hydrophobicity of the H-region promotes protein translocation [14–24]. However, a few mutagenesis studies of heterologous protein production showed that increasing the signal peptide hydrophobicity did not improve the yields [25–29].

The above studies did not account for the translational strengths of signal peptides. The nucleotide sequence of the signal peptide overlaps with the translation initiation region (TIR) which starts immediately upstream of the Shine-Dalgarno sequence and extends to around 20 nucleotides downstream of the initiation codon [30]. Changes in the TIR sequence can greatly affect secretion and periplasmic levels of heterologous proteins [31]. Changes to amino acid residues in the N-terminal portion of the signal sequence can alter the translation strength and make it challenging to evaluate the underlying cause of observed effects.

To address this problem, we controlled the translational strength of various signal peptides by silent mutagenesis and analyzed their effects on the production of full-length mAbs. Our results demonstrated that under conditions of similar translational strength, the hydrophobicity of the signal peptide is critical for heavy chain secretion to the periplasm. Increasing hydrophobicity of the signal peptide enhanced the periplasmic levels of heavy chain and further increased the yield of assembled full-length mAbs.

## Results

### Production of full-length hu5D5 in the periplasm of *E. coli* and challenges in antibody chain secretion

**Fig. 1:**
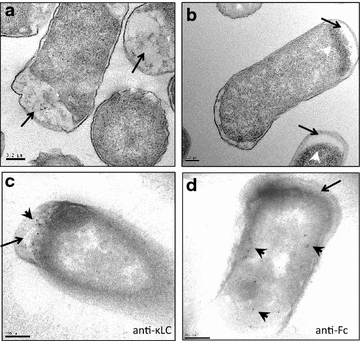
Electron microscopy of *E. coli* expressing full-length hu5D5. **a**–**b** Transmission electron microscopy (TEM) images of 64B4 host strain expressing the empty vector pBR322 (**a**) or pBR-ssSTII0.3-ssSTII1-hu5D5 (**b**). *White arrowheads* point to inclusion bodies and *black arrows* point to the periplasm. **c**–**d** Immunostaining EM probing for light chain using anti-LC antibody (**c**) or heavy chain using anti-Fc antibody (**d**). *Black arrows* point to the periplasm. *Black arrowheads* point to *gold particles* indicating the cellular localization of light chain or heavy chain. *Scale bars* equal to 200 nm. The complete plasmid sequences were confirmed by DNA sequencing

We started with a humanized anti-MET hu5D5.v2 antibody [32] (referred to as hu5D5 in this study) as a model IgG1 to study the production of full-length mAb in the periplasm of *E. coli*. A two-cistron expression vector [33] was used, in which hu5D5 light chain and heavy chain sequences were each inserted downstream of the *phoA* promoter, *trp* Shine-Dalgarno sequence, and the signal sequence of Heat-Stable Enterotoxin II (ssSTII). Simmons et al. previously constructed ssSTII variants with various TIRs [31] and showed that lower TIR strengths resulted in increased full-length mAb production [33]. Based on this study, we used an ssSTII variant with a relative TIR strength of 0.3 (ssSTII0.3) for the light chain and an ssSTII variant with a relative TIR strength of 1 (ssSTII1) for the heavy chain. The DNA sequences and relative TIR strengths of the signal sequences are listed in Additional file 1: Table S1. The corresponding amino acid sequences are listed in Table 1. The expression vector was transformed into a periplasmic protease-deficient host strain 64B4 (Additional file 2: Table S2) and antibody chain expression was induced upon phosphate depletion in the shake flask culture. *E. coli* cells expressing hu5D5 formed large inclusion bodies in the cytoplasm (Fig. 1b). *E. coli* expressing the empty vector did not form inclusion bodies (Fig. 1a). This observation suggested inefficient secretion of hu5D5 light and/or heavy chain to the periplasm. Immunogold electron microscopy showed that hu5D5 light chain predominantly localized in the periplasm whereas heavy chain localized in the cytoplasm (Fig. 1c, d), indicating hu5D5 heavy chain may be trapped in the cytoplasm.

**Table 1:** The amino acid sequences of the signal peptide TIR variants

Signal peptide TIR variants	Amino acid sequences
ssSTII1 and ssSTII0.3	MKKNIAFLLASMFVFSIATNAYA
ssDsbA1	MKKIWLALAGLVLAFSASA
ssPhoA1	MKQSTIALALLPLLFTPVTKA
ssMalE1	MKIKTGARILALSALTTMMFSASALA
ssSTII1 S-13L	MKKNIAFLLA*L*MFVFSIATNAYA
ssSTII1 S-13I	MKKNIAFLLA*I*MFVFSIATNAYA
ssSTII1 S-13Y	MKKNIAFLLA*Y*MFVFSIATNAYA
ssSTII1 A-18L A-14L S-13L	MKKNI*L*FLL*LL*MFVFSIATNAYA
ssDsbA1 L-9I	MKKIWLALAG*I*VLAFSASA
ssDsbA1 L-9S	MKKIWLALAG*S*VLAFSASA
ssDsbA L-9A	MKKIWLALAG*A*VLAFSASA
ssDsbA L-9Q	MKKIWLALAG*Q*VLAFSASA

Amino acid substitutions are marked in italics

To confirm that the secretion of hu5D5 heavy chain was limited, we examined the appearance of the precursor and mature antibody chains. The non-secreted precursor form of an antibody chain maintains the signal peptide at the N-terminus. During protein translocation, the signal peptide is cleaved and therefore the secreted mature chain no longer contains the signal peptide sequence [34, 35]. For hu5D5 light chain, the precursor and mature forms can be distinguished by apparent molecular weight differences on SDS-PAGE. No precursor of light chain was detected using SDS-PAGE followed by Western blot analysis probing for light chain. N-terminal Edman sequencing showed that the signal sequence was partially detected, however, the yield of the amino acids was too low to be quantified (data not shown). For the hu5D5 heavy chain, N-terminal Edman sequencing was used to detect the precursor and mature forms because the small difference in molecular weight made it challenging to separate the two forms via SDS-PAGE. Interpretation of the sequencing data showed that less than half of the total amount of heavy chain was secreted (Table 2). To eliminate the possibility that light chain co-expression might interfere with heavy chain secretion, we expressed heavy chain with the STII signal sequence in the absence of light chain, and did not observe an increase in heavy chain secretion efficiency (Table 2). In contrast, no precursor was detected when hu5D5 light chain was expressed alone (Additional file 3: Figure S1). Taken together, these results demonstrated that the secretion of hu5D5 heavy chain, but not the light chain, was limiting.

**Table 2:** The processing of hu5D5 heavy chain mediated by ssSTII, ssDsbA, ssPhoA, and ssMalE at a relative TIR strength of one

	Signal peptide	Mature HC %
LC and HC co-expressed	ssSTII1	45
	ssDsbA1	86
	ssPhoA1	6
	ssMalE1	35
HC only	ssSTII1	29
	ssDsbA1	84
	ssPhoA1	4
	ssMalE1	12

### The effect of various signal peptides on hu5D5 heavy chain secretion to the periplasm

In order to improve the secretion of hu5D5 heavy chain to the periplasm, we tested signal peptides representing two major bacterial secretion pathways: the signal sequence of DsbA (ssDsbA), which mediates co-translational secretion and is dependent upon the signal recognition particle (SRP) [36]; and the signal sequences of the maltose binding protein MalE and alkaline phosphatase PhoA (ssMalE, ssPhoA), which mediate sec-dependent post-translational secretion [37–40]. To control the translational strengths, we utilized silent mutations to generate signal peptide variants with various TIRs (see “Methods” section). ssSTII, ssDsbA, ssPhoA, and ssMalE TIR variants with relative translational strengths of ~1 (Additional file 1: Table S1, named as ssSTII1, ssDsbA1, ssPhoA1, ssMalE1) were each separately fused to hu5D5 heavy chain and tested for their ability to mediate hu5D5 secretion to the periplasm in the absence of light chain.

N-terminal Edman sequencing revealed that ssSTII1, ssMalE1, and ssPhoA1 resulted in more precursor than mature heavy chain, whereas ssDsbA1 led to more mature heavy chain than precursor (Table 2), whether light chain was co-expressed or not. Collectively, with a TIR strength of ~1, ssDsbA showed increased secretion efficiency for hu5D5 heavy chain compared to ssSTII, ssPhoA, and ssMalE.

### The effect of signal peptide hydrophobicity on hu5D5 heavy chain secretion at TIR strength 1

**Fig. 2:**
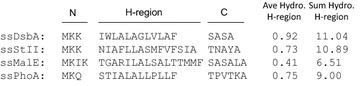
The amino acid sequences of signal peptides and the hydrophobicity of the H-region. The N-terminus, H-region, and the C-terminus were assigned following previous literature [35, 42, 53, 60]. The average and sum hydrophobicity of the H-region were calculated using the Eisenberg scale [61]

We sought to understand the molecular mechanism of why ssDsbA mediated more efficient secretion of hu5D5 heavy chain compared to the other signal peptides tested. Most *E. coli* signal peptides are composed of three distinct regions: an N-terminal region which contains one or two positively charged amino acid residues, a hydrophobic core region often referred to as the H-region, and a C-terminal region recognized by the signal peptidase [12, 13]. A comparison between ssSTII and ssDsbA showed that they have the same N-terminal residues and small residues Ala at the -1 and -3 positions of the cleavage site, suggesting that the N- and C-termini may not be the key factors that cause different secretion efficiency. Hydrophobicity calculation of the four signal peptides revealed that the H-region of ssDsbA is more hydrophobic than that of ssSTII, ssMalE, and ssPhoA (Fig. 2). Moreover, studies of multiple signal peptides suggest the hydrophobicity of the H-region is important for protein secretion, although the TIR strengths of the signal peptides were not controlled in these studies [14, 16, 21, 41, 42]. Therefore, we hypothesized that modulating the hydrophobicity of the H-region and controlling the TIR strength could improve hu5D5 heavy chain secretion.

To test this hypothesis, we introduced single amino acid changes into the H-region of ssSTII and ssDsbA (Table 3). The H-region of ssSTII contains two polar amino acid residues, Ser-13 and Ser-8. To increase the H-region hydrophobicity, Ser-13 was mutated to the residue Leu or Ile; as a control Ser-13 was also mutated to the non-charged polar residue Tyr, which has hydrophobicity similar to Ser (Table 3). Ser-8 was not changed due to the concern that it might affect cleavage by the signal peptidase. The TIR strengths of ssSTII hydrophobicity variants were normalized to ~1 by wobble-codon silent mutations (Additional file 1: Table S1). In the absence of light chain co-expression, both S-13L and S-13I substitutions in ssSTII increased hu5D5 heavy chain secretion to the periplasm (Table 3). The control S-13Y did not increase the secretion (Table 3). We also engineered a highly hydrophobic ssSTII TIR1 variant by introducing three leucines into the H-region. ssSTII A-18L, A-14L, S-13L did not further improve the secretion of hu5D5 heavy chain above the results obtained for S-13L (Table 3), suggesting the effects of H-region hydrophobicity reached a plateau. Similar plateau effects were observed in a study that modulated the hydrophobicity of ssPhoA [20]. For ssDsbA, we generated variants with decreased hydrophobicity by mutating Leu-9 to Ala, Ser, or Gln (Table 3). All the variants have a TIR strength of one. In the absence of light chain, both L-9S and L-9Q resulted in a decrease in secreted hu5D5 heavy chain. L-9A appeared to have a more modest impact on the secretion of hu5D5 heavy chain (Table 3). As a control, replacing Leu-9 with the hydrophobic residue Ile did not affect hu5D5 heavy chain secretion compared to the native signal peptide sequence (Table 3). We observed similar effects of signal peptide hydrophobicity on hu5D5 heavy chain secretion when light chain was co-expressed (Table 3). Collectively, at a TIR strength of 1, the hydrophobicity of the H-region is a critical factor for the efficient secretion of hu5D5 heavy chain.

**Table 3:** The effects of signal peptide hydrophobicity on hu5D5 heavy chain processing

	Signal peptide	ΔHydro	Mature HC %	Signal peptide	ΔHydro	Mature HC %
HC only	ssSTII1	–	29	ssDsbA1	–	84
	ssSTII1 S-13L	+1.24	70	ssDsbA1 L-9S	−1.24	38
	ssSTII1 S-13I	+1.56	58	ssDsbA1 L-9Q	−1.91	20
	ssSTII1 S-13Y	+0.44	22	ssDsbA1 L-9A	−0.44	60
	ssSTII1 A-18L A-14L S-13L	+2.12	65	ssDsbA1 L-9I	+0.32	82
LC and HC co-expressed	ssSTII1	–	45	ssDsbA	–	86
	ssSTII S-13L	+1.24	67	ssDsbA1 L-9S	−1.24	39
	ssSTII S-13I	+1.56	66	ssDsbA1 L-9I	+0.32	81

ΔHydro represents the change in total hydrophobicity resulting from the amino acid variants

### Improving assembled full-length hu5D5 levels by modulating signal peptide hydrophobicity at TIR strength 1

**Fig. 3:**
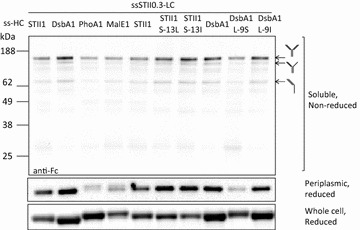
The effects of signal peptide hydrophobicity on full-length hu5D5 production. For all the constructs, light chain was fused to ssSTII0.3 and heavy chain was fused to ssSTII1, ssDsbA1, ssPhoA1, ssMalE1, ssSTII1 S-13L, ssSTII1 S-13I, ssDsbA1 L-9S, or ssDsbA L-9I. *Top panel* the non-reduced soluble protein samples from the cell lysates were separated by electrophoresis followed by Western blot analysis. Heavy chain-containing species are marked based on the apparent molecular weight. *Middle panel* Western blot of soluble heavy chain in the periplasm. The periplasmic protein samples were reduced with 0.2 M DTT. *Bottom panel* Western blot of reduced total heavy chain in the whole cell lysates. All the *blots* were probed with the HRP-conjugated anti-Fc antibody

We next asked if the hydrophobicity of the H-region also affects assembled full-length hu5D5 accumulation in the periplasm. To test this idea, the hu5D5 light chain was fused to ssSTII0.3 and hu5D5 heavy chain was fused to ssSTII1, ssDsbA1, ssPhoA1, ssMalE1, or the H-region hydrophobicity variants of ssSTII1 and ssDsbA1. Levels of soluble hu5D5 heavy chain and full-length hu5D5 in the periplasm were examined by Western blot analyses (Fig. 3). ssDsbA1 resulted in more soluble hu5D5 heavy chain and assembled full-length hu5D5 accumulation in the periplasm than ssSTII1, ssPhoA1, or ssMalE1. Decreasing the hydrophobicity of the ssDsbA1 H-region by using L-9S resulted in decreased levels of soluble heavy chain and assembled full-length hu5D5 in the periplasm (Fig. 3). As a control, ssDsbA L-9I did not affect the periplasmic accumulation of soluble heavy chain and assembled full-length hu5D5. For ssSTII, increasing the hydrophobicity of the native STII signal peptide by using S-13L or S-13I increased the accumulation of heavy chain and assembled full-length hu5D5. We did not observe any correlation between H-region hydrophobicity and total levels of hu5D5 heavy chain in the cells. However, the hu5D5 heavy chain migrated at a higher apparent molecular weight for ssMalE1, ssPhoA1, and ssDsbA L-9S. N-terminal sequencing confirmed that the major components of these bands were heavy chain precursors (Tables 2, 3). Collectively, the H-region hydrophobicity of the signal peptide plays an important role in full-length hu5D5 production, probably by increasing the amount of heavy chain secreted to the periplasm.

**Fig. 4:**
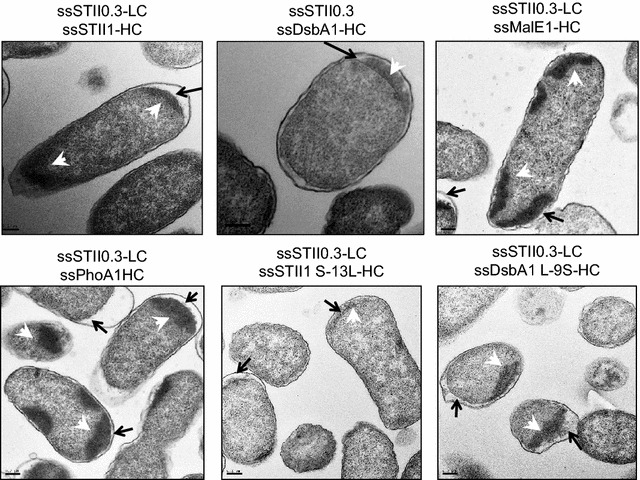
The effects of signal peptide hydrophobicity on the cellular localization of inclusion bodies. TEM of *E. coli* expressing pBR-ssSTII0.3-ssSTII1-hu5D5, pBR-ssSTII0.3-ssDsbA1-hu5D5, pBR-ssSTII0.3-ssMalE1-hu5D5, pBR-ssSTII0.3-ssPhoA1-hu5D5, pBR-ssSTII0.3-ssSTII1 S-13L-hu5D5, or pBR-ssSTII0.3-ssDsbA1 L-9S-hu5D5. *Black arrows* point to the periplasmic space, and the *white arrowheads* point to the inclusion bodies

H-region hydrophobicity also influenced the cellular localization of inclusion bodies (Fig. 4). Signal peptides with a less hydrophobic H-region fused to hu5D5 heavy chain (ssSTII1, ssPhoA1, ssMalE1, or ssDsbA1 L-9SS) resulted in inclusion body formation in the cytoplasm, suggesting that proteins were trapped in the cytoplasm and aggregated. In contrast, signal peptides with a more hydrophobic H-region (ssDsbA1 and ssSTII S-13L) resulted in more inclusion bodies in the periplasm, indicating that proteins were secreted to the periplasm and aggregated. Strategies to optimize antibody folding and prevent aggregation may further improve full-length hu5D5 production.

### The effects of signal peptide hydrophobicity on the production of other full-length mAbs

To test if the effects of signal peptide hydrophobicity apply to other full-length mAbs, two additional IgG1s (designated as mAb1 and mAb2) were included in this study. We first examined the secretion of mAb1 heavy chain and mAb2 heavy chain in the absence of their corresponding light chain. ssDsbA1 was found to mediate more efficient secretion of both mAb1 and mAb2 heavy chain compared to ssSTII1. Increasing the hydrophobicity of ssSTII1 with S-13L increased the secretion efficiency of both heavy chains, whereas decreasing the hydrophobicity of DsbA1 by L-9S had the opposite effect (Table 4). However, the impact of L-9S on the secretion of mAb1 heavy chain was greater than for mAb2 heavy chain (Table 4).

**Table 4:** The effects of signal peptide hydrophobicity on the secretion of mAb1 and mAb2 heavy chain

Signal peptide	Cargo protein	Mature HC %
ssSTII1	mAb1HC	35
ssSTII1 S-13L	mAb1HC	79
ssDsbA1	mAb1HC	86
ssDsbA1 L-9S	mAb1HC	13
ssSTII1	mAb2HC	17
ssSTII1 S-13L	mAb2HC	78
ssDsbA1	mAb2HC	89
ssDsbA1 L-9S	mAb2HC	72

**Fig. 5:**
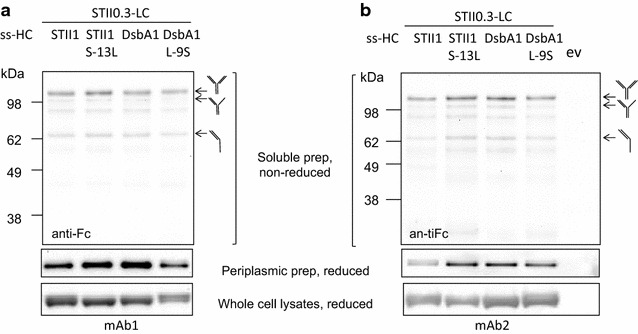
The effects of signal peptide hydrophobicity on full-length (**a**) mAb1 and (**b**) mAb2 production. For all the constructs, light chain was fused to ssSTII0.3 and heavy chain was fused to either ssSTII1, ssSTII1 S-13L, ssDsbA1, or ssDsbA1 L-9S. 64B4 harboring the empty vector (ev) pBR322 was loaded as the negative control. *Top panel* the non-reduced soluble protein samples from the cell lysates were separated by electrophoresis followed by Western blot probed with HRP-conjugated anti-Fc antibody. Heavy chain-containing species are marked based on the apparent molecular weight. *Middle panel* Western blot of soluble heavy chain in the periplasm. The periplasmic protein samples were reduced by 0.2 M DTT. *Bottom panel* Western blot of reduced total heavy chain in the whole cell lysate

To examine the effects of signal peptide hydrophobicity on the production of full-length mAb1 and mAb2, ssSTII0.3 was used for light chain and ssSTII1, ssDsbA1, ssSTII1 S-13L, or ssDsbA1 L11S for heavy chain. We observed for mAb1 that although ssDsbA1 led to more soluble heavy chain accumulation than ssSTII1, it did not lead to increased amounts of assembled full-length mAb (Fig. 5a). The use of ssSTII1 S-13L increased levels of both soluble heavy chain and assembled full-length mAb1 in the periplasm, and use of ssDsbA L-9S decreased the levels of soluble heavy chain and assembled full-length mAb1 (Fig. 5a). However, the differences in mAb1 levels were not as dramatic as those seen for hu5D5. For mAb2, the more hydrophobic signal peptides ssDsbA1 and ssSTII1 S-13L resulted in higher levels of soluble heavy chain and assembled full-length mAb in the periplasm than the less hydrophobic signal peptides ssSTII1 and ssDsbA1 L-9S (Fig. 5b). These results suggest that modulation of the signal peptide hydrophobicity impacts the accumulation of heavy chain and can impact the assembly of full-length mAb1 and mAb2. It is important to note that other factors such as antibody assembly efficiency and light chain-heavy chain interactions may limit the levels of full-length mAbs in the periplasm.

## Discussion

Production of aglycosylated full-length mAbs in *E. coli* has been previously demonstrated by secreting the light and heavy chain to the periplasm where the antibody fragments are assembled [33, 43, 44]. However, the secretory production of mAbs often faces challenges such as protein aggregation, proteolytic degradation, and inefficient secretion [3]. In this study we found that inefficient secretion of heavy chain can potentially limit the assembled full-length mAb level in the periplasm. We were able to improve heavy chain secretion and mAb accumulation by using a signal peptide with hydrophobic H-region at a controlled translational strength. The signal peptide engineering technique presented here may also apply to improve secretory production of other heterologous proteins in bacteria and to expand the range of proteins that can be efficiently presented by phage display [45].

Two aspects were considered for signal peptide engineering. First, we introduced amino acid substitutions to increase the hydrophobicity of the H-region, an important factor for protein secretion. Second, because amino acid changes in signal sequences alter the TIR sequence and potentially change the translational strength, and because high translational strength can result in inefficient secretion of the target protein [31], we used silent mutations that change the wobble base pairs in the signal sequence to control the translational strengths. Many previous studies on signal peptide mutagenesis showed that increasing signal peptide hydrophobicity improved protein translocation [14–24], whereas a few studies showed the opposite results [25–29]. The different observations may be due to the use of different signal peptides and/or cargo proteins in these studies. However, it is also important to note that these studies generally did not control the translational strength.

In *E. coli*, the majority of secreted proteins are targeted to the SecYEG translocon through two pathways: the SRP-dependent co-translational pathway and the SRP-independent post-translational pathway. In the co-translational pathway, SRP binds co-translationally to the newly synthesized signal peptide at the ribosome and directs the ribosome-nascent chain (RNC) complex to the SRP receptor FtsY. The RNCs are then targeted to the SecYEG translocon where translocation occurs simultaneously with translation. In the post-translational pathway, the polypeptide is released from the ribosome as the precursor form prior to translocation. The precursor is then targeted to the membrane-bound SecA and SecYEG in the inner membrane (reviewed in [1]). Although post-translational signal peptides have been extensively used for recombinant protein production, they may not be a good choice for recombinant proteins that fold or aggregate rapidly in the cytoplasm [46]. Such issues could potentially be prevented by using SRP-dependent signal sequences. With the co-translation pathway protein translation, translocation, and folding are coordinated. Among the signal peptides tested, ssDsbA mediates co-translational secretion [36], while ssPhoA and ssMalE have been shown to mediate post-translational secretion [37, 40]. Indeed, we observed that ssDsbA led to more efficient secretion of heavy chain than the two post-translational signal peptides (Table 2). Lee et al., also recently reported that using the DsbA signal sequence to replace the PelB signal sequence enhanced the production of full-length IgG [43]. It is unclear if ssSTII mediates co-translational or post-translation secretion. The observation of a large percentage of precursor heavy chains suggests that ssSTII preferentially uses the post-translational pathway, although it does not rule out the possibility that ssSTII mediates heavy chain secretion through both the post- and co-translational pathways. It has been shown that some proteins use both pathways for secretion [34, 47]. Moreover, recombinant protein expression and secretion can result in exceeding the capacity of one secretion pathway. In such a scenario it is possible that protein secretion utilizes both pathways.

How does signal peptide hydrophobicity affect heavy chain secretion? One possible explanation is that a less hydrophobic signal peptide such as ssSTII preferentially uses the post-translational pathway. The heavy chain precursors are likely to fold or aggregate in the cytoplasm and become secretion incompetent. Increasing the H-region hydrophobicity could re-route heavy chain to the co-translational secretion pathway and prevent heavy chain folding or aggregation in the cytoplasm. This hypothesis is supported by studies showing that hydrophobic signal peptides interact with SPR more efficiently [48–50] and often mediate co-translational secretion [24, 51]. Alternatively, it is possible that increasing the signal peptide hydrophobicity results in more efficient interaction between the heavy chain precursor and SecA [48] or cytoplasmic chaperones.

In addition to signal peptide hydrophobicity and TIR strengths, other factors can also impact antibody chain secretion. For instance, a positively charged amino acid at the N-terminus of the signal sequence has been shown to promote the interaction with SRP [52]. The alpha-helix content in the signal sequence is also important for efficient secretion [53, 54]. The amino acid composition of C-terminus is critical for the cleavage of signal peptide [35]. Moreover, several studies have shown that signal peptides can modulate the folding, thermodynamic stability, and aggregation propensities of cargo proteins [55, 56]. With the post-translational secretion pathway, interactions between the signal peptide and heavy chain in the cytoplasm may affect secretion efficiency.

Using signal peptide engineering, we increased the yields of hu5D5 in the shake flask cultures approximately 2.5–3 fold. Many factors remain to be tested to further optimize full-length mAb accumulation in the periplasm. For example, co-expression of key components of the secretion machinery may further increase antibody chain secretion. It’s been shown that over-expression of SRP (Ffh) increased the yields of full-length IgG [43], presumably due to increased secretion efficiency. Also, based on our preliminary results we suspect that heavy chain and light chain may compete for the limited secretion capacity. Therefore, the ratio of light chain to heavy chain may need to be finely tuned. Moreover, secreted light chain and heavy chain can form dimeric species or aggregates in the periplasm (Figs. 3, 5). Co-expression of periplasmic chaperones provides a feasible strategy to prevent protein aggregation and increase yields of mAbs [8, 43, 44].

## Conclusions

*Escherichia coli* is an attractive vehicle for the production of therapeutic proteins. However, it remains challenging to achieve high yields for complex, multi-subunit proteins including full-length mAbs. In this study we demonstrated that one of the bottlenecks for the production of assembled full-length mAbs is the secretion of heavy chain to the periplasm. Signal peptide engineering by controlling the TIR strength and increasing H-region hydrophobicity improved the efficiency of heavy chain secretion to the periplasm and further improved the accumulation of several full-length mAbs. The technology described here offers an effective approach for production of full-length mAbs in *E. coli*.

## Methods

### Strains and plasmids

Strains and plasmids used in this study are listed in Additional file 2: Table S2. To construct expression vectors, DNA fragments of heavy chain or light chain containing the *phoA* promoter and signal sequence were cloned into a pBR322-derived expression vector [33]. To construct *phoA* reporter plasmids for translational strength measurement, the fragments containing the signal peptide sequences were cloned upstream of the *phoA* reporter gene in the pPhoA86 vector [31]. Additional site-specific mutations in signal peptide sequences were introduced by the QuickChange Site-Directed Mutagenesis Kit (Stratagene). The sequence of each plasmid was confirmed by DNA sequencing (Genentech Inc.).

### Construction of signal peptide TIR libraries

Libraries of signal sequences with a range of translational strengths were generated as described previously [31, 57]. Briefly, degenerate oligos were used to introduce silent mutations in the first seven codons of signal sequences from the*stII*, *dsbA*, *malE*, and *phoA*. Restriction sites of *XbaI*, *BssHII*, or *MluI* were inserted upstream of the start codon to diversify the TIR region. The TIR variant sequences were inserted upstream of the mature *phoA* gene on the pPhoA81 plasmid. The constructs were transformed into the host strain 27C7. The transformants were plated on LB agar plates containing carbenicillin and 100 µg/mL of the substrate 5-bromo-4-chloro-3-indolyl phosphate (BCIP, Sigma) and grown at 37 °C overnight. Colonies with light blue or dark blue color were selected for plasmid extraction and sequencing (Genentech, Inc.).

### Translational strength measurement

The translational strengths of the signal peptide variants were determined by the alkaline phosphatase assay using para-nitrophenylphosphate as the substrate. [31, 57].

### Induction of antibody expression

Bacterial cultures were grown in Luria–Bertani (LB) or complete C.R.A.P. phosphate-limiting media [33] containing antibiotics at 37 °C or at 30 °C as indicated. The following concentrations of antibiotics were used: carbenicillin 50 µg/mL, and tetracycline 20 µg/mL. For protein expression, the host strain 64B4 harboring the antibody expression vector was inoculated into 5 mL of LB supplemented with 20 µL/mL tetracycline and 5 mM sodium phosphate, pH 7 in a polypropylene culture tube and incubated at 30 °C with shaking overnight. OD_550_ of the overnight culture was measured to ensure the strain did not have growth defects. 0.5 mL of the overnight culture was inoculated into 25 mL of complete C.R.A.P. supplemented with 20 µL/mL tetracycline in a 125 mL baffled shake flask and cells were grown at 30 °C with shaking for 24 h. The optical density of the culture at 7 and 24 h was measured at 550 nm.

### Protein extraction and Western blotting analysis

End point samples from shake flask cultures were collected for Western blot analysis. To measure total heavy chain levels, 1 mL of 1 OD_550_ of bacteria was spun down, lysed in 200 µL tricine SDS buffer with 0.2 M dithiothreitol (DTT, Sigma), and heated at 95 °C for 5 min.

To extract soluble proteins, a previously described sonication approach [57] was used because it resulted in more reproducible results and higher enrichment of antibody chains compared to the conventional osmotic shock method. Briefly, whole cell broth was diluted into chilled lysis buffer (10 mM Tris, pH 6.8, 5 mM EDTA, 0.2 mg/mL Lysozyme, and 5 mM iodoacetic acid) to a final OD_550_ of 3.0. 600 µL of samples were then sonicated using two rounds of 10 × 1 s pulses and centrifuged for 15 min at 16,000 ×g at 4 °C. Supernatant was collected for SDS-PAGE and Western blot analysis. For periplasmic protein extraction, 10 OD_550_ of bacteria were pelleted and resuspended in TBS buffer (200 mM Tris, pH8.0, 0.5 mM sucrose, 1 mM EDTA) with 1 tablet of protease inhibitor cocktail (Roche) as described [58]. Soluble protein samples or periplasmic extracts were mixed 1:1 (v/v) with tricine SDS buffer with or without 0.2 M DTT and then analyzed by SDS-PAGE and Western blotting. Lane intensities on coomassie stained gels were used as a loading control. Heavy chain-containing species were probed with HRP-conjugated goat anti-human Fc antibody (Pierce). Light chain-containing species were probed with HRP-conjugated goat-anti-human kLC antibody (Bethyl Laboratories). Target proteins on immunoblots were detected by enhanced chemiluminescent (GE Healthcare).

### Edman sequencing of the N-terminus of heavy chain

End point samples from shake flask cultures were normalized to 4 OD_550_ and harvested by centrifugation at 16,000×*g* for 3 min. Whole cell lysates resuspended in 200 µL tricine SDS buffer containing 0.2 M DTT were loaded onto 10 % Bis–Tris SDS-PAGE. After electrophoretic separation, the proteins were transferred to PVDF membranes using wet transfer (Biorad) in CAPS buffer. The heavy chain band at ~50 kDa on the membrane was excised and subjected to Edman sequencing analysis using the Applied Biosystems Procise Sequencer Model 494HT. Picomole values of each amino acid were calculated by the sequence analysis program SEQX normalized against the uncorrected phenylthiohydantoin amino acid standards [59]. An average of 10 cycles was used to produce the repetitive yield plot to calculate the linear regression, and the initial yields of major and minor sequences were defined as the y-intercepts of the plotted lines. The heavy chain processing efficiency was calculated as the percentage of mature heavy chain, which equals *Initial yield*_*mature*_*/(Initial yield*_*mature*_ + *Initial yield*_*precursor*_*)*.

### Transmission electron microscopy (TEM)

End point samples from shake flask cultures were immediately collected, pelleted to 50–100 µL pellets, and resuspended in 1 mL of in the modified Karnovsky’s fixative (2 % paraformaldehyde and 2.5 % glutaraldehyde in 0.1 M sodium cacodylate buffer, pH7.2) and then post-fixed in 1 % aqueous osmium tetroxide (EM Sciences, Hatfield, PA) for 1 h followed by overnight incubation in 0.5 % uranyl acetate at 4 °C. The samples were then dehydrated through a series of increasing ethanol concentrations (50, 70, 90, 100 %), followed by propylene oxide (each step was for 15 min) and embedded in Eponate 12 (Ted Pella, Redding, CA). Ultrathin sections (80 nm) were cut with an Ultracut microtome (Leica), stained with 0.2 % lead citrate and examined using a JEOL JEM-1400 transmission electron microscope (TEM) at 120 kV. Digital images were captured with a GATAN Ultrascan 1000 CCD camera.

### Immunogold electron microscopy (immunoEM)

For the immunogold EM experiments, samples were first prepared for cryosectioning. The cells were fixed in 4 % paraformaldehyde with 0.1 % glutaraldehyde in phosphate buffer (0.1 M, pH 7.2), washed several times in PBS, embedded in 12 % gelatin and infiltrated in 2.3 M sucrose overnight at 4 °C. Samples were then mounted on pins for cryo-ultramicrotomy frozen in a cryosectioning chamber (supplied with liquid nitrogen). Ultrathin cryosections (100 nm) were prepared with a diamond knife (Diatome) at −80 °C using an ultramicrotome (Ultracut; Leica) equipped with a cryosectioning chamber. Thawed cryosections were transferred to Formvar- and carbon-coated EM grids (Nickel) with a drop of 2.3 M sucrose, immunolabeled (see below) and then counterstained for EM with 0.5 % uranyl acetate in 2 % methylcellulose for 1 min at room temperature. For immunogold labeling, the thawed cryosections on grids were blocked in blocking agent (Aurion Inc) for 30 min and incubated with an anti-light chain (LC) or anti-heavy chain (Fc) HRP-conjugated goat antibody for 45 min at room temperature, followed by incubation with an anti-HRP gold conjugated goat antibody (Jackson ImmmunoResearch) for 30 min. Sections were then counterstained as described above. Immunogold-labeled sections were visualized and examined in a JEOL JEM-1400 transmission electron microscope (TEM) at 120 kV. Digital images were captured with a GATAN Ultrascan 1000 CCD camera.

## Additional files

10.1186/s12934-016-0445-3 The DNA sequences of the signal peptide TIR variants and the relative TIR strengths.
10.1186/s12934-016-0445-3 Strains and plasmids used in this study.
10.1186/s12934-016-0445-3 Western blot analysis of light chain secretion.
