# Supplementary material for: Enhancing full-length antibody production by signal peptide engineering
Source: Microb Cell Fact. 2016 Mar 2;15:47. doi: 10.1186/s12934-016-0445-3 (PMC4776426; doi:10.1186/s12934-016-0445-3)

Figure S1: Western blot analysis of light chain secretion. hu5D5 light chain was fused to ssSTII1, ssDsbA1, ssPhoA1, or ssMalE1. Only light chain was expressed and heavy chain was not co-expressed. The whole cell lysates were reduced and probed with HRP-conjugated anti-kappa light chain antibody.

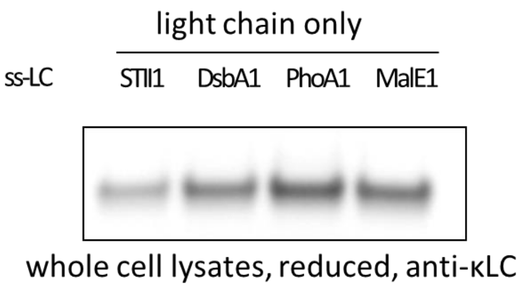

Supplement: Supplementary file 3 — 10.1186/s12934-016-0445-3 Western blot analysis of light chain secretion. [file 12934_2016_445_MOESM3_ESM.pdf]
